# Online GIS services for mapping and sharing disease information

**DOI:** 10.1186/1476-072X-7-8

**Published:** 2008-02-25

**Authors:** Sheng Gao, Darka Mioc, Francois Anton, Xiaolun Yi, David J Coleman

**Affiliations:** 1 Department of Geodesy and Geomatics Engineering, University of New Brunswick, Canada; 2 Department of Informatics and Mathematical Modelling, Technical University of Denmark, Denmark; 3 New Brunswick Lung Association, Canada

## Abstract

**Background:**

Disease data sharing is important for the collaborative preparation, response, and recovery stages of disease control. Disease phenomena are strongly associated with spatial and temporal factors. Web-based Geographical Information Systems provide a real-time and dynamic way to represent disease information on maps. However, data heterogeneities, integration, interoperability, and cartographical representation are still major challenges in the health geographic fields. These challenges cause barriers in extensively sharing health data and restrain the effectiveness in understanding and responding to disease outbreaks. To overcome these challenges in disease data mapping and sharing, the senior authors have designed an interoperable service oriented architecture based on Open Geospatial Consortium specifications to share the spatio-temporal disease information.

**Results:**

A case study of infectious disease mapping across New Brunswick (Canada) and Maine (USA) was carried out to evaluate the proposed architecture, which uses standard Web Map Service, Styled Layer Descriptor and Web Map Context specifications. The case study shows the effectiveness of an infectious disease surveillance system and enables cross-border visualization, analysis, and sharing of infectious disease information through interactive maps and/or animation in collaboration with multiple partners via a distributed network. It enables data sharing and users' collaboration in an open and interactive manner.

**Conclusion:**

In this project, we develop a service oriented architecture for online disease mapping that is distributed, loosely coupled, and interoperable. An implementation of this architecture has been applied to the New Brunswick and Maine infectious disease studies. We have shown that the development of standard health services and spatial data infrastructure can enhance the efficiency and effectiveness of public health surveillance.

## Background

Currently, such factors as booming population, environmental pollution, rapid urbanization, and global warming all influence the conditions for disease outbreaks. Disease studies have revealed strong spatial aspects, including disease case location and disease diffusion. Thus, mapping spatial aspects of diseases could help people understand some puzzles of disease outbreak. The development of disease mapping was traced by Tom Koch from a map of plague outbreaks at Bari, Italy in 1694 to a map of AIDS for the entire earth in the present-day [1]. Unlike the raw disease data, disease maps offer a visual means of identifying cause and effect relationships existing between humans and their environment. Disease maps can enable health practitioners and the general public to visually communicate about disease distribution.

Geographical Information Systems (GIS) provide an effective way of managing, storing, analyzing, and mapping disease information. GIS has strong capabilities in mapping and analyzing not only spatial data, but also non-spatial data, and can integrate many kinds of data to greatly enhance disease surveillance. It can render disease data along with other kinds of data like demographic and environmental data, representing the differences with various cartographical styles. Gupta and Shriram [2] identified many useful functions of GIS such as network analysis, buffer analysis, and statistical analysis in the area of disease surveillance. When a disease appears, GIS could represent disease information rapidly and analyze the disease's spread dynamically. Boulos [3] emphasized that the GIS technologies and services that are able to function proactively in real time are extremely and critically important to creating a "spatial health information infrastructure".

Meanwhile, the rapid development of the Internet influences the popularity of Web-based GIS, which itself shows great potential for the sharing of disease information through distributed networks. Distributing and sharing disease maps via the Web could help decision makers across health jurisdictions and authorities collaborate in preventing, controlling and responding to a specific disease outbreak.

Documented applications are already making health information accessible through the Web [4,5]. Custom online interactive health maps could be implemented using Google Maps API, Google Earth KML or MSN Virtual Earth Map Control [6]. The maturity of Web-based GIS enables the generation of thematic maps dynamically and efficiently, with a thin/thick client or hybrid architectures. For example, Inoue et al. [7] developed a thin client, Web-based GIS application to dynamically generate and display infectious disease surveillance data through maps and charts. Blanton et al. [8] integrated federal, state and local data and developed map tools for rabies surveillance with a Web-based GIS thin client architecture. Other applications have employed thick client, Web-based GIS approaches to visualize health information through Java Applets or Scalable Vector Graphics (SVG). Qian et al. [9] provided a thick client, Web-based GIS approach to visualize global SARS information using a Java Applet. Kamadjeu and Tolentino [10] implemented a Web-based public health information system to generate district-level country immunization coverage maps and graphs with SVG. As the response performance of Web-based GIS is in near real time, it is effective for understanding the disease phenomena to support decision making.

Time is an important factor in analyzing disease outbreak. Foody [11] highlighted the spatio-temporal characteristic as an important feature in recent health studies. By comparing the thematic maps at different time intervals, the spatial-temporal change of the disease could be projected, including temporal cluster shift, vector transmission rates, and mobility of susceptible populations. Greene et al. [12] analyzed the spatial, temporal and spatio-temporal patterns of viral meningitis to aid the identification of risk factors. Greiling et al. [13] developed a desktop application with a time bar for exploring spatio-temporal patterns of colon cancer mortality rates.

### Challenges in disease mapping

The experience of disease outbreak has demonstrated the importance of applying statistical models and mapping tools in making health policies. Despite the continual development of disease mapping technologies, four major challenges still exist.

#### 1. Disease data heterogeneities

Disease data are collected by different health organizations in various ways, which creates a barrier to data sharing. These data may be stored and distributed in different places through files or databases. Commonly, there are three sources of heterogeneity: semantic, syntactic, and schematic heterogeneities that need to be considered during data integration [14]. Techniques that can facilitate the sharing and integration of disease data are highly valuable. *Semantic heterogeneity *arises from the cognitive differences and naming convention variations among various disciplines. *Schematic heterogeneity *deals with the different methods of describing the facts of the world, including hierarchies, properties, and relationships. *Syntactic heterogeneity *refers to diversity in representations or storage models. The schema integration approach and ontology-based approach could be used to overcome these heterogeneities and thus facilitate data sharing.

#### 2. Difficulties in integration and reusability

Integrating and reusing the current health applications is constrained to a large extent. Zeng et al. [15] pointed out that the isolation of existing stand-alone disease management systems leads to a data sharing problem. Most of the health information systems have a closed architecture – even the ones that use Web-based technology are difficult to integrate. Typically, users can only access maps from such a health application, and it is difficult to integrate datasets from these applications. A service oriented architecture with loosely coupled services could link distributed health data and support reuse of services.

#### 3. Lack of interoperability between different disease services

Interoperability makes it easy to communicate, execute programs, or transfer data among various systems in a unified manner. For disease studies, it is important to utilize distributed disease information and share the data through standard interfaces. In analyzing disease information and the health decision making process, it is helpful to integrate many kinds of spatial and non-spatial data, including roads, hospitals, available medical resources, etc. To address spatial data sharing and interoperability, many international organizations such as Open Geospatial Consortium (OGC), and the International Standards Organization Technical Committee 211 (ISO/TC211) are attempting to address standards and application specifications. Since spatial representation makes disease phenomena more understandable, integrating these open spatial standards for the development of Web-based disease tracking and analysis systems represents a great opportunity to improve health data sharing, interoperability and visualization. Boulos and Honda [16] proposed to publish the health maps through Open Source Web GIS software that usually supports OGC specifications.

#### 4. Concerns over appropriate cartographical representation and sensitive dissemination of disease data

Cartographical representation deals with the data representation in graphics. It greatly influences the understanding of disease phenomena. Many health practitioners are eager to map the disease data to certain district boundaries, which could show the patterns of disease distribution and support their decision making. Disease data contain private information, and sharing of such data may cause considerable concern. For example, if the disease information shows one area with high disease rates, people would possibly avoid both the area and its inhabitants. Bell et al. [17] listed four kinds of methods to protect the confidentiality of disease data: (a) the aggregation of data in spatial and temporal dimensions; (b) removal of the geographical identifiers from the original data; (c) relocation of individual records randomly on a small scale; and (d) limitation of access to the data through a user- and/or function-restricted computer environment. When compared with original data, the aggregated results would have some differences. Leitner and Curtis [18] identified geographic masking methods used to preserve individual confidentiality and measured the similarity of the aggregated data through different cell sizes with the original point pattern. Meanwhile, such factors as population density, racial tendency, environmental pollution, and cultural difference all affect disease studies. Considering those factors in the mapping process will improve the cartographic representation of disease information.

## Methods

### Disease mapping architecture

**Figure 1:**
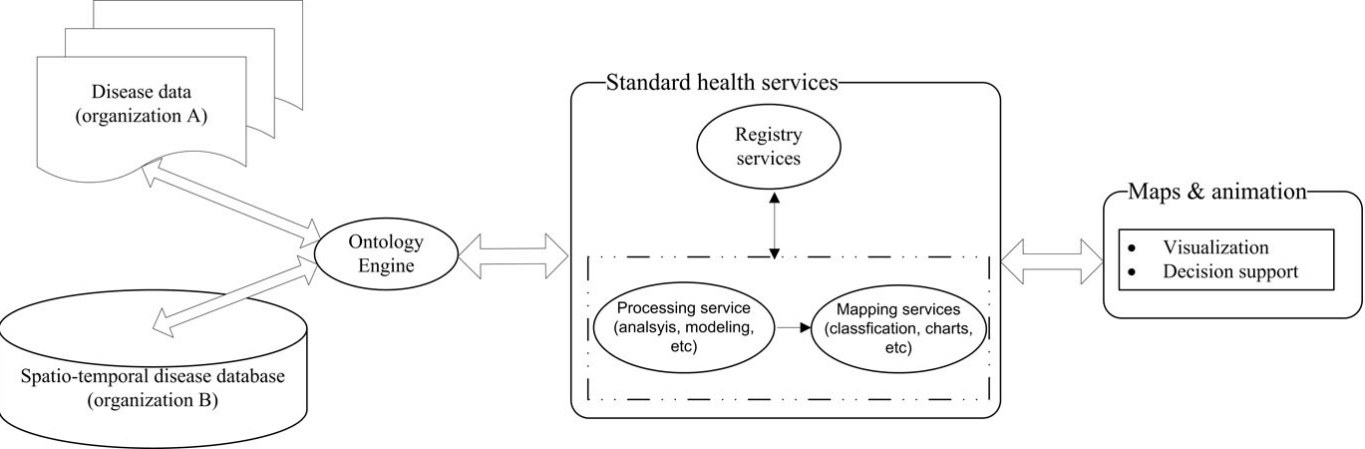
**Disease mapping architecture**. This architecture includes a data storage tier, an ontology engine tier, a standard health services tier, and a maps and animation tier.

To overcome in particular the heterogeneous data integration and service interoperability challenges to disease mapping, we propose the disease mapping architecture illustrated in Figure 1. The architecture contains four tiers: a *data storage *tier, an *ontology engine *tier, a *standard health services *tier, and a *maps and animation *tier.

#### • Data storage tier

The health data could be collected by different health organizations and stored in files or databases. They can be accessed through the Internet or Intranet for data sharing.

#### • Ontology engine tier

The ontology engine is designed to overcome the heterogeneities existing in the distributed health data. It provides a uniform way for the standard health services to retrieve data. Health data matching and transformation tasks are processed by the ontology engine.

#### • Standard health services tier

Explicit standards are proposed to be used in this tier for the interoperability of the disease mapping system. OGC provides many specifications in sharing spatial related data, which is possible to support disease data sharing. Generally, there would be three kinds of services:

- *Health data processing services *are responsible for analyzing the disease from spatial and temporal aspects. Many statistical methods are used in the analysis of the disease. Most common ones are crude morbidity ratio, and standardized morbidity ratio. Other methods use spatial autocorrelation indicators like Moran's I and Local G* in detecting disease clusters [13].

- *Health mapping services *could serve the cartographical representation of the health data to the clients. Providing disease information through dynamically generated maps could control privacy issues more effectively than the SVG or Java Applet technologies which transfer the disease data to the client side.

- *Health registry services *act as the service brokers in the service-oriented architecture. With the health registry services, all the description information about health processing services, and health mapping services could be published and discovered conveniently through uniform interfaces.

#### • Maps and animation tier

It provides the spatio-temporal maps for the health practitioners and public in their decision making process. Ogao [19] categorized three types of animation methods from "low" to "high" according to the respective levels of interactivity and complementary domain knowledge that each of them offers to the user: passive, interactive and inference-based animations. Through visualization tools like maps and animation, people could generate hypotheses in disease studies and seek the explanatory factors, which is important in decision making. The ability to share the maps or animations in a distributed environment could also provide a collaborative mechanism in preparation, response, and recovery stages of disease control.

### Study area and data description

The province of New Brunswick, Canada and the state of Maine, U.S.A. are our study areas. They share a common, highly travelled territorial border. There are significant volumes of goods and people travelling across this international border and infectious agents are easily carried across both sides. To assure the privacy of the health data, different health organizations or users have different rights in accessing detailed levels of the health data. There will be different levels of privilege in dealing with visualizing and tracking the levels of health data.

In this study, we choose six levels of administrative/census areas that cover the entire territory of both sides. New Brunswick is organized into "Province", "Health Region", "Census Division", "Census Subdivision", "Forward Sortation Area" and "Dissemination Area" geo-layers. In Maine, the corresponding levels are "State", "Health Service Area", "County", "County Subdivision", "Zip Code" and "Census Block Group" respectively.

The data for infectious disease mapping used in this study includes disease data, population data and six levels of geometric boundary data. The infectious disease data for New Brunswick are represented by the hospital discharge data recorded for the New Brunswick Department of Health between 1997 and 2002. The corresponding Maine data were collected through our research partners at the University of Southern Maine. The six levels of geometric boundary data for New Brunswick were obtained from Service New Brunswick, Statistics Canada and Canadian Geospatial Data Infrastructure (CGDI) portal. The six levels of geometric boundary data for Maine were obtained from the American National Spatial Data Infrastructure (NSDI) portal.

The population data of New Brunswick and Maine were acquired from Statistics Canada and the U.S. Census Bureau respectively.

### Spatial-temporal data model and data matching

**Figure 2:**
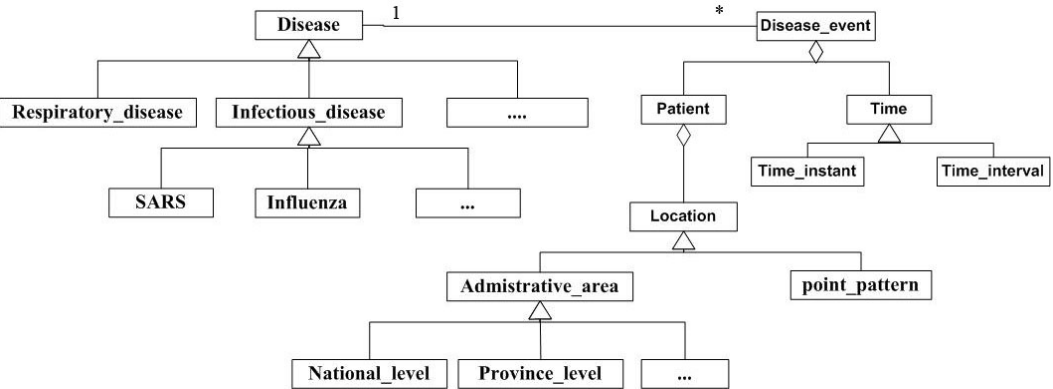
**Spatio-temporal data model for disease data**. This data model is an object-oriented model and used for the data integration.

The spatio-temporal object-oriented data model can provide a uniform way to manage spatio-temporal data and support better data management and analysis. The spatio-temporal object-oriented data model used in this study is shown in Figure 2. The *Disease class*, which describes the disease characteristics, could be extended to its subcategories of disease such as *Infectious disease *and *Respiratory disease*. By comparision, a *Disease event *is a spatio-temporal object that relates to certain kind of disease. It is the activity that associates with a certain kind of disease, such as a hospital observation, training and education service to patients. It includes the patient and the time information. *Time *could be an instant or interval. *Patient *is related to the disease case location. *Location *could be administrative area or geo-coding point. *Administrative area *could be national level, provincial level, county level, etc.

We integrate the data from New Brunswick and Maine mainly through a common schema integration approach. All the attributes in describing disease, disease event, patient, time, and the six administrative geographic levels of both sides are specified. For instance, in constructing the jurisdiction of Health Region, common attributes such as name, spatial boundary, state/province code and vaccine stock are described. Moreover, a data dictionary is built to match the similar world facts with different definitions to the common schema. For example, the postal code attribute used in New Brunswick and zip code attribute used in Maine are matched to the postcode attribute in the common schema. Through the data matching, the Maine data and New Brunswick data would then be handled in the same way.

### Statistical methods for data processing

In this study, we concentrated on the spatial, temporal, and demographic factors and their influence on the infectious disease outbreak, which could show the disease distribution with spatial, temporal, age and gender differences. The statistical methods used are basic statistical calculations of disease rates, as more complex methods would delay the response time in the online mapping process. These statistical methods are the following: Crude Morbidity Rate (CMR), Normalized Morbidity Ratio (NMR), Age-Specific Morbidity Ratio (ASMR), Age-Adjusted Morbidity Ratio (AAMR), and Standardized Morbidity Ratio (SMR).

The purpose of these statistical methods is to provide a standardized legend (pattern/colour) for data representation across temporal, spatial, and jurisdictional layers. The disease data used are in point patterns, which are generated through geo-coding process with the postal code and/or geo-coded civic addresses. Since the name of the postal code may change over time, we consider the spatial location of postal code and/or geo-coded civic addresses to ensure the geo-coding quality. With the "point-in-polygon" spatial operation, it is easy to roll up data and calculate disease cases in relation to certain administrative boundaries. The above five statistical methods are used to calculate the statistical values of disease rates. These statistical values could be expressed through disease mapping variables related to time (e.g., annual, seasonal, monthly, weekly, daily), gender (e.g., male, female, both), age group (e.g., 0–4, ..., 85+, total), geographic level (e.g., Dissemination Areas/Census Block Group, Census Divisions/County, etc.), and/or disease type (e.g., influenza). In the classification maps or charts, the generated thematic maps are based on the above multiple disease mapping variables.

Processing time is also an important factor for online infectious disease mapping, as it takes time to calculate the statistical values. Taking this into account, we have developed two flexible interfaces for obtaining the statistical results. For precomputed cases, the system could respond in real time. In such a case, the statistical values of the pre-defined conditions (spatial level, age group, etc) have already been calculated. The other situation is more flexible and is processed in real time. Users can define the parameters (certain time interval, specific age group, etc) according to their requirements. In addition, a cache mechanism is developed to maintain calculated statistical values. Data warehousing can be used as an alternative approach to improve the processing performance.

### OGC services for disease mapping

The OGC Web Map Service (WMS), Styled Layer Descriptor (SLD), and Web Map Context (WMC) are implemented for the disease mapping and sharing in this study. WMS publishes its ability to produce maps rather than its ability to access specific data holdings, and generates spatially referenced maps dynamically [20]. SLD allows user-defined symbolization in producing maps [21], which make it possible to integrate maps from different WMS in the same style. WMC uses eXtensible Markup Language (XML) based context documents including information about the servers providing layers in the overall map, the bounding box, and map projection shared by all the maps, and these provide sufficient operational metadata for clients to reproduce the maps [22].

## Results

**Figure 3:**
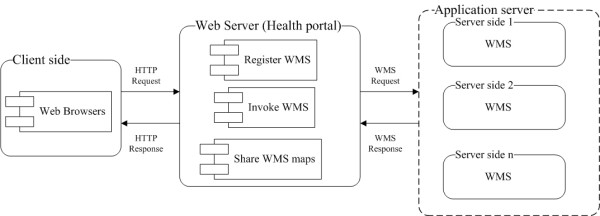
**Implemented mapping and collaboration framework**. The framework contains client side, health portal and application server.

This study deals with the visualization of infectious disease spatio-temporal outbreaks and propagation across New Brunswick and Maine in different resolutions, through the implementation of a service oriented online infectious disease mapping and sharing system. The implemented framework is shown in Figure 3. All the WMS services could be registered in the health portal for user access. Through the health portal, users could obtain disease maps from the desired WMS that distributes over the Internet, and share the acquired WMS maps with others through WMC.

### Web Map Service support

The most important operation in the Web Map Service is GetMap. It supports the parameters for getting images in certain spatial extent, time, coordinate reference system, style, image height, image width, and image format. To maintain the flexibility of showing the maps in different styles, SLD supports user-defined symbolization in representing the data in maps. For instance, multiple disease maps accessed from different WMS Services can be represented using the same cartographical style.

**Figure 4:**
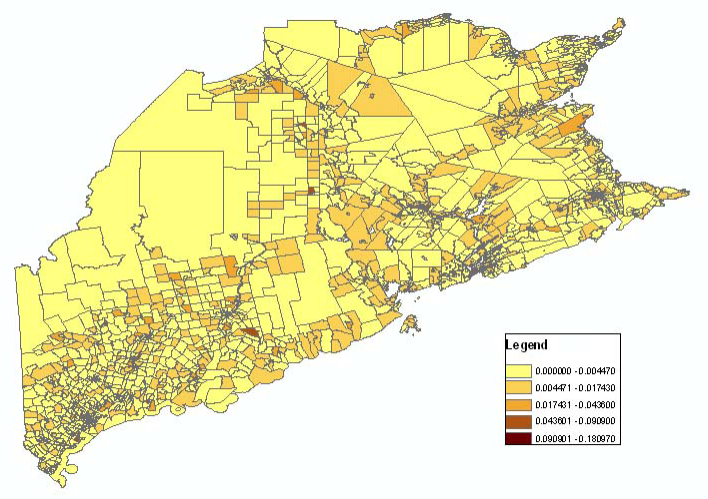
**Crude Morbidity Ratio 2000**. It represents Crude Morbidity Ratio distribution of all the cells with the parameters (Dissemination Area/Census Block Group level, year 2000, all age group, influenza).

**Figure 5:**
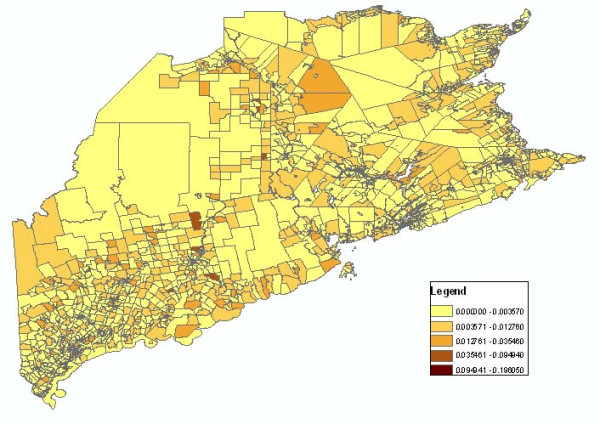
**Crude Morbidity Ratio 2001**. It represents Crude Morbidity Ratio distribution of all the cells with the parameters (Dissemination Area/Census Block Group level, year 2001, all age group, influenza).

In the infectious disease mapping process, several mapping variables, including age group, statistical method, and gender need to be considered. However, the standard Web Map Service could not support parameters such as disease type, gender, and statistical method, among others. For the integration of web map services in the disease mapping, we developed a convention to name map layers. As to different combinations of gender, age, geographic level, disease type and statistical method variables, we assign a distinct WMS layer name to each of them through customized encoding rules. The web map service parses the infectious disease mapping parameters from the layer name. As the service is compatible with WMS, thematic disease maps could be accessed by a health portal or any OGC compatible clients. Figure 4 shows the classification map retrieved from a web map service which describes Crude Morbidity Ratio distribution of all the cells with the parameters (Dissemination Area/Census Block Group level, year 2000, Crude Morbidity Ratio, all age group, influenza). Figure 5 shows the Crude Morbidity Ratio distribution of year 2001 with the same parameters. By comparing different mapping variables at different times and geographical levels, the users can visualize the pattern and movement of the infectious disease.

**Figure 6:**
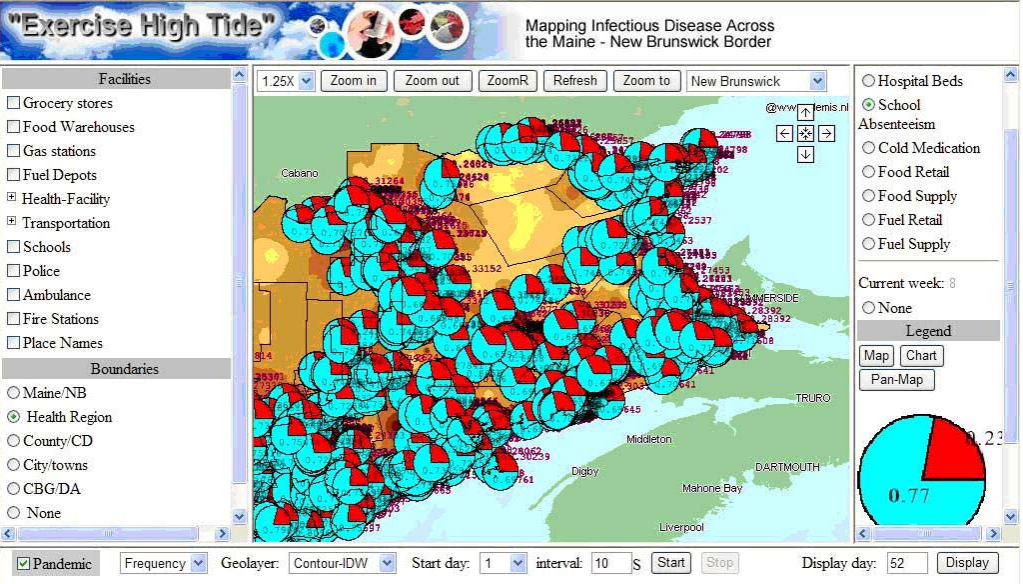
**Web Map Service integration**. It is integrated from three WMS services that produce school absenteeism charts, thematic disease maps and world boundary maps.

In addition, simulated influenza outbreak data (includes the influenza cases, other data such as grocery retail, grocery supply, fuel retail, fuel supply, school, pharmacy and hospital beds occupation) based on a 1968 influenza variation (approximately 35% infection rate) were generated and published through WMS. Hosted by the Emergency Measures Organization of the Province of New Brunswick, Exercise "High Tide" enlisted many participants to test this real-time decision making environment in the simulation of a disease outbreak. This environment simulates the diffusion of the disease at different days using animated mapping. The animation is achieved by using the time tag in WMS services. Users can select a start date and map switch interval to view the disease map animation, or choose a certain day to show the disease map. In the generation of the disease maps, this environment supports the data aggregation and representation to certain levels, such as Maine/NB and Health Region, in different days. With the specified user request, the mapping values in the database temporal tables (which stores the geometry data and mapping attribute values) will be updated synchronously with a lock mechanism and disease maps will be created. Meanwhile, the maps of facilities like grocery stores and ambulance locations could be obtained from a WMS. Figure 6 shows the school absenteeism chart obtained from a web map service on top of the thematic disease map, and the background image is also retrieved from a WMS provided by Demis, a European company. The convenient disease map access and integration could be achieved by using the standard WMS.

### WMC for sharing disease maps

**Figure 7:**
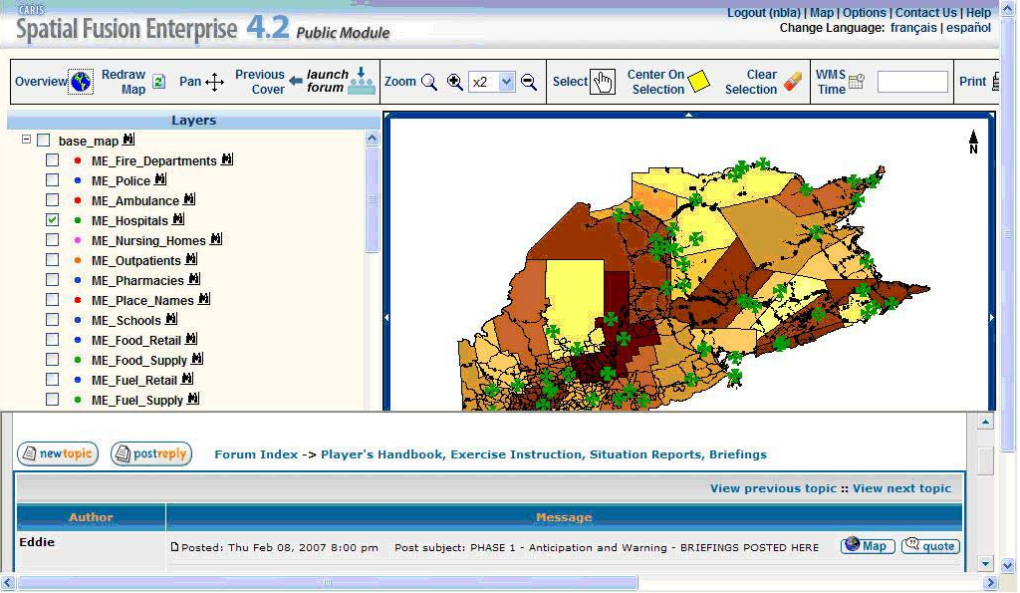
**Discussion forum for decision making**. After users click the "launch forum" button, they could log into the forum and share maps and text with others.

Collaboration is very important in disease decision-making. The sharing of disease maps allows users to readily discuss how to prepare for and respond to disease outbreaks. Following the previous work of developing an online GIS discussion forum for public participation [23,24], we integrate a discussion forum with CARIS Spatial Fusion Enterprise in a health portal, which can access and distribute disease maps from WMS. Compared with pure text, maps are more attractive in sharing certain types of ideas with others. The portal allows users to exchange ideas with text as well as maps (see Figure 7). The Spatial Fusion Enterprise is used for accessing the disease map from different WMS services. In addition to the ordinary forum functions, this forum provides the capacity to view disease maps and attach the current map view of Spatial Fusion Enterprise to a user's topic.

**Figure 8:**
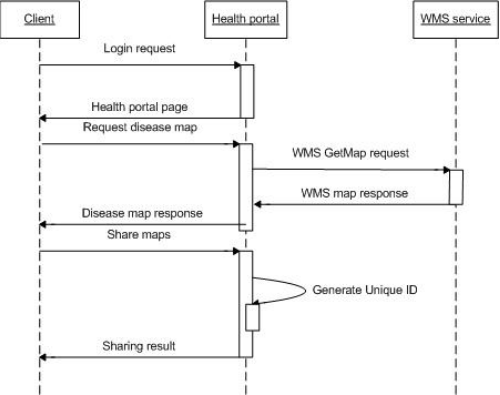
**Service level sequential diagram for disease data sharing**. After users log into the forum, they can obtain disease maps and share them with others. Each shared map is given a unique identification.

The service level sequential diagram of this system is shown in Figure 8. After the users log into the health portal, they can request the disease maps that they need in their application. The health portal will invoke the appropriate WMS and show disease maps to the users. If users want to share the maps, they can launch the discussion forum and attach the disease maps to a posted topic. The health portal would generate a unique ID to the shared disease maps and save the parameters rather than the maps in obtaining the disease maps through WMC. WMC stores the parameters in XML with general element for layer-independent context and a sequential layer list for specific details about each shared layer. Afterwards, when other people visit the forum and click the map button in a certain topic, the health portal will parse the according WMC document, obtain the disease maps, and show them in the viewer.

## Discussion

With the implementation of the standard service oriented disease mapping architecture, sharing disease data through the distributed network can achieve high flexibility and interoperability. The health services could be defined in fine granularity and composed into service chains for satisfying the requirements of different applications. In disease studies, health organizations could generate their own disease mapping and processing services compatible with OGC specifications and register them in a common catalogue. In this way, the cost of disease data collection and analysis can be shared. At the same time, the ability and options for collaboration have been greatly improved.

With the statistical methods for data processing, we can aggregate the disease data to certain levels to be mapped. The thematic maps and map animation are used to show the disease information and protect the confidentiality of disease data. Disease information cartographical representation is generated in this project based on health users' needs.

By proposing an OGC-compliant architecture to implement Web-based health services, the authors contend that the issues of reusability, integration and interoperability of services are well handled in this project. Moreover, the services could be enriched based on the continuous development of OGC specifications. Other OGC standard services – for example, Web Processing Service (WPS) for processing functions and Web Catalogue Service (WCAS) – will be implemented in future health applications.

Data heterogeneity problems always occur in the data collection process of different health organizations. This case study accomplishes a low-level integration by converting the data from both sides to a common schema. It solves schematic and syntactical heterogeneity issues, but does little to address semantic heterogeneity. Building a standard ontology for the spatio-temporal disease data would enable the concept-based sharing of disease data, solving the semantic heterogeneity problems (cognition and naming differences).

The senior authors are currently integrating a health model with the OGC geospatial data model in generating standard ontology to support better sharing and integration of disease data. The heterogeneous data integration process will be implemented in two phrases. After considering the semantic issues of the text information, spatial pattern and topology will then be incorporated into the integration.

## Conclusion

Recent disease outbreaks have demonstrated the need for GIS- and mapping-related applications in public health. The World Health Organization, American Centers for Disease Control, and Health Canada are all proactively engaged in mapping viral pandemics and applying GIS models to global and national health policy. In this research, we designed and implemented a service oriented online disease mapping architecture which is loosely coupled and interoperable. This architecture supports reusability of health disease data mapping and analysis functions to lower the cost of building huge independent disease surveillance systems. It also enables cross-border map visualization, analysis, and sharing disease information through interactive maps or animation in a collaborative manner with multiple partners (public health officials, researchers, policy-makers and the public) via a distributed network. If a real disease outbreak occurs, this distributed disease mapping architecture can support public education, disease surveillance, health care planning, emergency coordination, spatial epidemiology, vaccine distribution, and policy initiatives at different administrative levels. If the disease data can be updated frequently, health practitioners could obtain real-time disease maps processed in accordance with different statistical methods and under different spatio-temporal conditions in order to understand both the current situation and the movement of disease. More effective collaboration with the support of disease maps over the internet can secure a faster response to emergency situations. A case study of infectious disease mapping across New Brunswick and Maine has been implemented on the proposed architecture to cope with the disease data sharing, integration and representation challenges. More extensive implementation of standards-based Spatial Data infrastructure (SDI) in each country could enable effective collaborative decision making and policy planning. The development of SDI would further support this online disease mapping architecture for decision and policy making. To improve the effectiveness and efficiency of this architecture for disease applications, future research will concentrate on development of geospatial disease ontology to facilitate data integration and the construction of interoperable distributed disease services.

## Competing interests

The author(s) declare that they have no competing interests.

## Authors' contributions

All authors designed and implemented the overall architecture for disease data mapping and sharing. All authors read and approved the final manuscript.
